# Quorum Quenching Enzymes and Their Application in Degrading Signal Molecules to Block Quorum Sensing-Dependent Infection

**DOI:** 10.3390/ijms140917477

**Published:** 2013-08-26

**Authors:** Fang Chen, Yuxin Gao, Xiaoyi Chen, Zhimin Yu, Xianzhen Li

**Affiliations:** School of Biological Engineering, Dalian Polytechnic University, Dalian 116034, China; E-Mails: sanmiancxh@163.com (F.C.); yuxindl2006@163.com (Y.G.); cxyrain@hotmail.com (X.C.); yuzhimin2005@163.com (Z.Y.)

**Keywords:** quorum sensing, quorum quenching, lactonase, acylase, acyl homoserine lactone, specificity

## Abstract

With the emergence of antibiotic-resistant strains of bacteria, the available options for treating bacterial infections have become very limited, and the search for a novel general antibacterial therapy has received much greater attention. Quorum quenching can be used to control disease in a quorum sensing system by triggering the pathogenic phenotype. The interference with the quorum sensing system by the quorum quenching enzyme is a potential strategy for replacing traditional antibiotics because the quorum quenching strategy does not aim to kill the pathogen or limit cell growth but to shut down the expression of the pathogenic gene. Quorum quenching enzymes have been identified in quorum sensing and non-quorum sensing microbes, including lactonase, acylase, oxidoreductase and paraoxonase. Lactonase is widely conserved in a range of bacterial species and has variable substrate spectra. The existence of quorum quenching enzymes in the quorum sensing microbes can attenuate their quorum sensing, leading to blocking unnecessary gene expression and pathogenic phenotypes. In this review, we discuss the physiological function of quorum quenching enzymes in bacterial infection and elucidate the enzymatic protection in quorum sensing systems for host diseases and their application in resistance against microbial diseases.

## 1. Introduction

Quorum sensing (QS) is a signaling system that occurs in the pathogenic kingdom to sense its own population density and synchronize the expression of the virulence gene via the secretion of small, diffusible signal molecules, such as *N*-acyl-homoserine lactone (AHL), termed autoinducers [1]. Autoinducers play a critical role in triggering virulence gene expression in QS-dependent pathogens, such as in the production of rotting enzyme. Interfering with the microbial QS system by quorum quenching (QQ) has been suggested as a potential strategy for disease control [2] because QQ aims to shut down the virulence expression in pathogenic bacteria rather than restrict cell growth and has shown potential to overcome drug toxicities, complicated super-infection and antibiotic resistance [3]. The interest in enzymatic function for protecting against microbial infection has intensified in recent years. QQ enzymes have been identified in a number of bacteria that have shown considerable promise as quorum quenchers since AHL-lactonase AiiA was first identified from *Bacillus* species to attenuate virulence in *Erwinia carotovora* [4].

QQ can be developed as a technique for disrupting the ability of a pathogen to sense its cell density and disable or diminish the capability of triggering the virulent expression. This capability ensures that the host has time to eradicate the pathogens naturally through normal immune system function, resulting in overcoming the pathogenic infection. Because it is different from conventional antibiotic therapy, which kills bacteria by interfering with DNA, RNA or protein synthesis, leading to the emergence of antibiotic-resistant superbugs, QQ is a promising approach that may lead to the development of very effective next generation antibacterial drugs based on interfering with bacterial communication to block QS-mediated pathogenic infection.

The physiological function of most QQ enzymes is not consistently clear, but these enzymes are found in QS and non-QS microbes [4]. Co-culturing the QQ producer with QS-dependent microbial pathogens has been shown to attenuate QS related activities. This study aimed to elucidate the enzymatic protection for host diseases in the QS system and its application in resistance against microbial diseases, primarily focusing on (1) the biodiversity of organisms with the potential to quench the QS signals; (2) the enzymatic degradation of the QS signals by QQ enzymes; (3) the function and characteristics of the enzymes with QQ activity; (4) the molecular phylogenesis of QQ enzymes in the QS system; (5) the enzymatic degradation of global signal molecules in the cell-cell signal transduction pathway; and (6) the application of antimicrobial agents of enzymatic protection in controlling microbial disease by interfering with the QS system, which expounds on the degradation of the autoinducers to block microbial attack. It is important that the enzymatic protections are completely non-disruptive to the environment and that their use will lead to a reduction in the application of chemicals.

## 2. Biodiversity of Organisms with Potential to Quench QS Signals

A number of bacterial cells produce and respond to AHLs that are small and diffusible signals involved in cell-to-cell communication. Bacteria can sense their population density by the concentration of signal molecules and coordinate their behavior, for instance to release toxins synchronously [5]. In addition to the QS inhibitor, the degradation of the QS signal by the QQ enzyme is another promising method of controlling microbial disease [6]. Since the AHL-degrading enzyme was first identified in *Bacillus* species, the QQ mechanism has been identified in many prokaryotic and eukaryotic organisms [7]. QQ has been shown to regulate the microbial activities of host by interfering with bacterial QS [8].

Over the last decade, many microbes capable of degrading QS molecules have been documented; the first report of such degradation was the isolation of *Bacillus* sp. 240B [2]. The strain 240B can produce lactonase, cleave the lactone ring from the acyl moiety of AHLs and render the AHLs inactive in signal transduction. The expression of the *aiiA* gene encoding AHL-lactonase in transformed *Pectobacterium carotovorum* has been shown to significantly reduce the formed QS molecule AHL, thereby quenching potato soft-rot by *Pec. carotovorum*. Soon after, the QQ microbes *Variovorax paradoxus* and *Ralstonia* sp. XJ12B were isolated, which are able to secrete the AHL-degrading enzyme with acylase activity [9,10]. Subsequent database searches for the homologues of the QQ enzyme in complete bacterial genomes have shown the existence of related enzymes in a wide range of species. Most of the characterized microbes are spread among the QS-mediated pathogens, and a few data related to AHL-degradation are from non-QS bacteria [2,9–11]. Many efforts are being invested to search for potential quorum quenchers and their roles in the QS-mediated mechanism in pathogens.

QQ microbes have been identified in a range of Gram-negative and Gram-positive microorganisms. The strains capable of degrading AHL by lactonase have been reported for *Bacillus* [12–14], *Agrobacterium* [15], *Rhodococcus* [16], *Streptomyces* [17], *Arthrobacter* [18], *Pseudomonas* [19] and *Klebsiella* [15]. The strains with acylase activity have been identified in *Pseudomonas* [20,21], *Ralstonia* [10,22], *Comamonas* [23], *Shewanella* [24] and *Streptomyces* [17].

QQ enzymes have been found in many species of the genus *Bacillus* since *Bacillus* sp. 240B was first found to be involved in the QS-QQ system. Broad-spectrum AHL-degrading AiiA enzymes were found to be widespread in the *B. thuringiensis* and *B. cereus* strains [12,13]. Recently, *B. amyloliquefaciens*, *B. subtilis*, *B. mycoides* and *B. marcorestinctum* were shown to have AHL-degrading activities, whereas AHL-degrading activity has not yet been reported for *B. pseudomycoides* [11,12,25,26]. AHL-degrading *B. sonorensis* L62 was isolated from a sample of the fermentation brine of Chinese soy sauce [27], which efficiently degraded *N*-(3-oxohexanoyl)- homoserine lactone (3-oxo-C6-HSL) and *N*-octanoyl-homoserine lactone (C8-HSL). The *aiiA* homologue was not detected in *B. sonorensis* L62, suggesting the presence of a different AHL-degrading gene in the strain L62.

In addition to *Bacillus* spp., a wide variety of bacteria have been shown to have QQ capability, including *A. tumefaciens* producing AttM and AiiB [28,29], *Arthrobacter* producing AhlD [18], *K. pneumonia* producing AhlK [18], *Ochrobactrum* producing AidH [30], *Microbacterium testaceum* producing AiiM [31], *Solibacillus silvestris* producing AhlS [32] and *Rhodococcus* strains W2, LS31 and PI33 producing QsdA [16,33]. *Chryseobacterium* strains isolated from the plant root have been shown to degrade AHL, and some strains showed putative AHL-lactonase activity, although the ability to degrade AHL is not a functional trait of the *Chryseobacterium* genus [34]. Yoon *et al.* [35] and Kang *et al.* [36] isolated AHL-degrading *Nocardioides kongjuensis* and *Acinetobacter*, which can hydrolyze AHL autoinducers, from soil samples. An endophytic Gram-positive *M. testaceum* was isolated from potato leaves and showed AHL-degrading activity [37]. Chen *et al.* [38] recently reported that *R. solancearum* GMI1000 produced a putative aculeacin A acylase with distinct QQ activity. *Comamonas* exhibited a wide range of AHL degradative patterns, varying with acyl chain lengths between four and 16 carbons with or without C3 substitutions [23]. Among the species harboring QQ enzyme activity, *Rh. erythropolis* is remarkable because it is the only bacterium in which three enzymatic activities directed at AHLs have been identified, including an oxidoreductase, amidohydrolase and lactonase [16,22,33].

*Ralstonia* strain XJ12B can degrade and grow rapidly on short- and long-chain AHLs [10], but *V. paradoxus* utilizes the entire range of short- and long-chain AHLs and grows most rapidly on 3-oxo-C6-HSL. The *P. aeruginosa* strain 2SW8 is similar to *V. paradoxus* and can utilize 3-oxo-C6-HSL as the sole energy source [39]. In *P. aeruginosa*, the acylase PvdQ has been identified as a late responder to the 3-oxo-C12-HSL QS circuit [20]. Subsequently, another *quiP* gene encoding acylase was identified, but it was not required for AHL utilization in the identical strain [40]. AHL-lactonase activity was recently found in *Acinetobacter* sp. [41].

In studying the ecosystem of the tobacco rhizosphere, Uroz *et al.* [42] isolated 25 strains responsible for the degradation of the QS signal molecule AHLs. Those isolates were categorized into six groups according to their genomic REP-PCR and PCR-RFLP profiles. The representative strains of the isolates were identified as members of the genera *Pseudomonas*, *Comamonas*, *Variovorax* and *Rhodococcus*. When the *Rh. erythropolis* strain W2 was used for quenching the QS-mediated microbial function, it strongly interfered with violacein production by *Chromobacterium violaceum* and markedly reduced the pathogenicity of the *Pec. carotovorum* subsp. *carotovorum* in potato tubers. This result revealed the diversity of the QS-interfering bacteria in the rhizosphere and the validity of targeting QS signal molecules to control pathogens with natural bacterial isolates.

**Figure 1:**
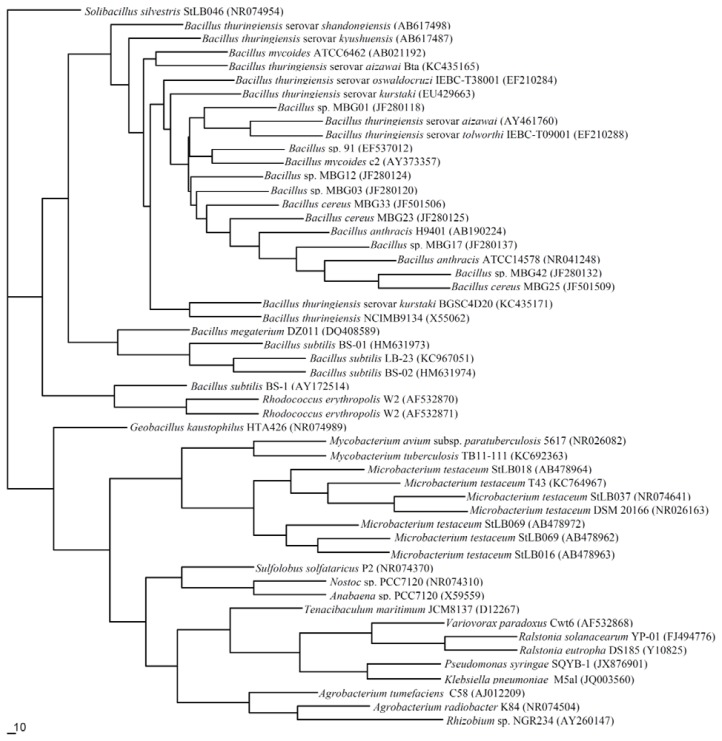
Neighbor-joining phylogenetic tree based on 16S rDNA gene sequences of quorum quenching (QQ) bacteria.

Based on 16S rDNA sequences retrieved from the GenBank database, the phylogenetic relationship for QQ bacteria was constructed in Figure 1. QQ bacteria can be divided into three phyla, including Firmicutes, such as *Bacillus* sp., *Geobacillus* sp. and *S. silvestris*, Actinobacteria, such as *Arthrobacter*, *M. testaceum*, *Rh. erythropolis*, *M. avium* and *Streptomyces*, and Proteobacteria, such as *Agrobacterium*, *V. paradoxus*, *R. solanacearum*, *Shewanella* sp., *P. aeruginosa*, *Comamonas* sp., *Burkholderia* sp., and *Acinetobacter* sp.. Most of the demonstrated genera that have the ability to quench QS enzymatically are α-Proteobacteria, including *Agrobacterium* and *Ochrobactrum* [28,43]; β-Proteobacteria, such as *Variovorax*, *Comamonas*, *Ralstonia*, *Delftia* and *Burkholderia* [9,10,42,43]; γ-Proteobacteria, such as *Pseudomonas*, *Acinetobacter* and *Shewanella* [20,36,42,44]; Gram-positive bacteria with low G + C, such as *Bacillus* [2,13,45]; and Gram-positive bacteria with high G + C, such as *Rhodococcus* [42].

The QQ enzymes occur both in non-QS (e.g., *B. marcorestinctum*) [11] and QS (e.g., *P. aeruginosa*) [19] microorganisms. The QS signal molecules typically were cleaved to form homoserine or a fatty acid and used as carbon and nitrogen sources for cell growth [20]. Most of the non-QS microbes responsible for the QQ activity were capable of metabolizing the QS single molecules, which likely evolved to utilize this commonly found QS signal molecule as a carbon and nitrogen source. One of the best-characterized soil bacterium, *V. paradoxus*, can use AHLs as a sole source of carbon and nitrogen [9].

In some cases, QS-bacteria could degrade their own autoinducers to terminate quorum-sensing activities. For example, *A. tumefaciens* produces AHL-lactonase AttM in the stationary phase that can degrade the *A. tumefaciens* autoinducer [28]. *E. carotovora* and *Xanthomonas campestris* show a similar loss of AHLs in the stationary phase [46,47]. The tropical marine *Pseudomonas* strain MW3A produces C12-HSL and 3-oxo-C14-HSL and degrades C6-HSL, 3-oxo-C6-HSL and 3-oxo-C8-HSL [48]. The QS-dependent microbe *Rhizobium* sp. strain NGR234 has two *traI* and *ngrI* loci linked to the synthesis of autoinducer I molecules, whereas it carries a large number of functional genes involved in AHL degradation [49]. At least five loci were detected in AHL degradation or modification. One of those enzymes showed similar activity to that of β-lactanase, and another resembled a bacterial dienelactone hydrolase; the remaining three loci encode a β-lactanase, an acetaldehyde dehydrogenase and a putative histidine triad protein linked with a predicted Nudix hydrolase.

## 3. Enzymatic Degradation of QS Signals by QQ Enzymes

**Figure 2:**
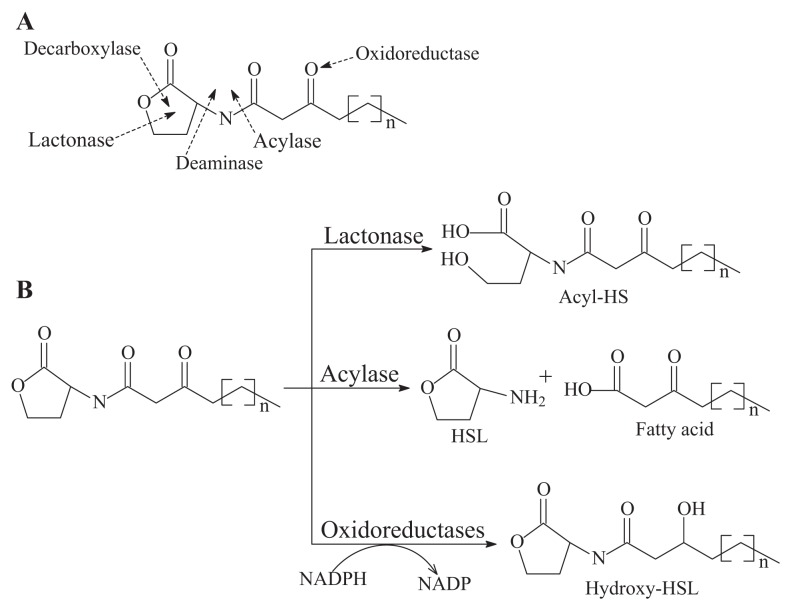
Possible linkage degraded by quorum quenching enzymes in quorum sensing molecule N-acyl homoserine lactone (**A**) and corresponding degradation mechanism of quorum quenching enzymes (**B**).

A limited number of QQ enzymes that interfere with bacterial QS molecules are known, although the general mechanisms of the QS system are well understood. It is presumable that four potential cleavage sites in the QS signal molecule AHLs are likely cut off enzymatically based on the AHL structure, as shown in Figure 2A, following a catabolic digestion of carbon and nitrogen sources by QQ microbes for cell growth. The enzymes catalyzing AHL degradation can be divided into two groups: one that leads to the degradation of the homoserine lactone ring mediated by lactonase or decarboxylase and one that causes the cleavage of AHL to a homoserine lactone and a free fatty acid moiety by acylase or deaminase. Only two enzyme families in the microorganism have the capability of cutting AHL structures; the AiiA-like AHL-lactonases and the AiiD-like AHL-acylases have been demonstrated to be involved in the real cleavage of the QS signal molecules for quenching QS, although a large diversity of QQ microbes have been identified [2,9]. The other two types of enzymes have not been identified. An oxidoreductase was included in quenching QS by substituting the oxo-group at C3 with the hydroxyl group, which may successively be degraded by amidohydrolase to form homoserine lactone and hydroxydecanoic acid [22]. Although the role of those QQ enzymes in their native environments is not always clear, their QQ ability and utility in potential industrial and therapeutic applications are promising.

The QQ enzyme can be grouped into two classes. Class I is defined as the enzyme-breaking AHL molecule, which contains AHL-lactonase, AHL-acylase and paraoxonase. Class II is the enzyme that reduces carbonyl to hydroxyl, which contains oxidoreductase.

**Figure 3:**
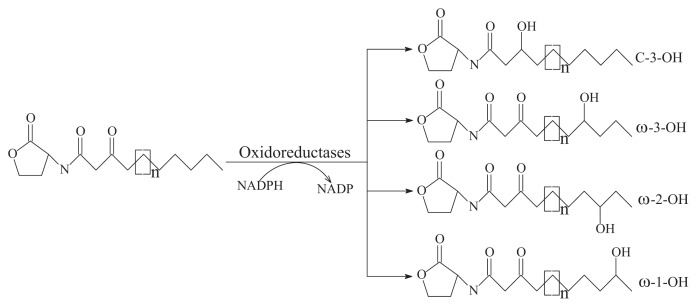
The quorum sensing (QS) signal molecule *N*-acyl-homoserine lactone (AHL) was reduced by oxidoreductases via substituting the oxo-group at the C3 or ω-1, ω-2 and ω-3 positions with hydroxyl group.

(i) AHL-lactonase cleaves the homoserine lactone ring of molecule AHLs in a hydrolytic and reversible manner to open the homoserine lactone ring, as shown in Figure 2B, which renders the QS molecule incapable of binding to the target transcriptional regulator and attenuates the effectiveness of the signal molecule [2]. Such hydrolysis is identical to pH-mediated lactonolysis and can be reversed by acidification. Two families of lactonases have been identified in prokaryotes according to their overall similarity and the original microbes. One of the well-studied families is represented by the AiiA lactonase metallohydrolase, which requires two Zn^2+^ ions for full functionality [50,51]. The AiiA-like lactonase activity is not affected by differences in the acyl chain length and substitution in the AHLs. The second type of AHL-lactonase is represented by the QsdA lactonase from the *Rh. erythropolis* strain W2, which is not related to the AiiA lactonase family, although both are Zn^2+^-dependent metalloproteins [33]. QsdA belongs to the phosphotriesterase family that harbors phosphotriesterase, lactonase or amidohydrolase activities [33] and is more closely related to the phosphotriesterase-like lactonases, such as SsoPox from *Sulfolobus solfataricus*, which has a perfectly fitting pocket where the lactone ring and acyl chain interact [52].
  The first known cluster was designated as AiiA in *Bacillus*, followed by AttM in *Agrobacterium* [2,15]. Recently, other types of lactonases, such as BpiB, AiiM and AidH, have been identified and extend the diversity of the lactonase family proteins [30,31,53]. All of these lactonases are Zn^2+^-dependent lactonases that occurred in the bacterial genera *Bacillus* [13], *Agrobacterium* [15], *Rhodococcus* [16], *Streptomyces* [17], *Arthrobacter* [18], *Pseudomonas* [19] and *Klebsiella* [18], except the lactonase derived from *Rhodococcus*, which forms a new family within the metal-dependent lactonases [33].
(ii) AHL-acylase irreversibly hydrolyzes the amide linkage between the acyl chain and homoserine moiety of AHL molecules. As shown in Figure 2B, this process releases homoserine lactone and the corresponding fatty acid, which do not exhibit any residual signaling activity [10]. The AHL-acylase was first described in the *V. paradoxus* strain VAI-C, which showed a wide range of AHL degradation capacity [9]. Subsequently, AHL-acylases from various groups of bacteria have been reported, predominantly including AiiD in *Ralstonia* sp XJ12B [10]; AhlM in *Streptomyces* sp. M664 [17]; PvdQ and QuiP in *P. aeruginosa* PAO1 [19,20,40]; and AiiC in *Anabaena* sp. PCC7120 [54]. Recently, an *aac* gene homologous to the AiiD acylase with undemonstrated function was identified from *R. solanacearum* GMI1000 [38]. Novel AHL-degrading genes have been isolated from the metagenomic libraries constructed from soil samples [50,53].
(iii) Oxidoreductase targets the acyl side chain by oxidative or reducing activities and thus catalyzes a modification of the chemical structure of the signal but not degradation, as shown in Figure 2B. Such modification might affect the specificity and recognition of the AHL signal, thus disturbing the activation of the QS-mediated genes regulated by a particular AHL [41]. Long-chained AHLs and fatty acids with varying chain lengths at various positions could be oxidized. Two types of oxidoreductases have been discovered. The P-450/NADPH-P450 reductase, a previously known enzyme with fatty acids as the substrate, has been isolated from *B. megaterium* CYP102A1 and characterized in detail [55]. This substrate is capable of the efficient oxidation of AHLs at the ω-1, ω-2 and ω-3 carbons of the acyl chain to eliminate their quorum sensing activity (Figure 3). This oxidation of AHLs represents an important and different QQ mechanism: breaking AHL molecules [2,10]. Uroz *et al.* [22] reported the presence of another enzyme in *Rh. erythropolis* W in which the 3-oxo substituent of 3-oxo-C14-HSL was reduced to yield the corresponding derivative 3-hydroxy-C14-HSL and the QS system was inactivated. Recently, a novel oxidoreductase BpiB09 derived from a metagenomic library was found to be capable of inactivating 3-oxo-C12-HSL [56]. Its expression in *P. aeruginosa* PAO1 resulted in significantly reduced pyocyanin production, decreased motility and poor biofilm formation, although AHLs are likely not the native substrate of this metagenome-derived enzyme.
(iv) The AHL-like-lactonases (paraoxonase) from mammalian sera have been described as AHL-lactonase-like enzymes and are involved in the hydrolysis of organophosphates [57].

## 4. Function and Characteristics of Enzymes with QQ Activity

The physiological function of those QQ enzymes and whether AHLs are their primary substrates have not been entirely clarified. Some characterized QQ enzymes are shown with their origin and substrate specificity in Table 1.

AHL-acylases display high substrate specificity based on the length of the AHL acyl chains in quenching QS signals [19], whereas the AHL-lactonases have a broader AHL substrate spectrum when inactivating AHL signal activity [18]. The AHL QuiP and PvdQ acylases are specific in 3-oxo-C12-HSL and are excellent examples that degrade only AHLs with long acyl-chains and not short acyl-chains [19]. AHL-acylase AiiD exhibits preference for the degradation of long-chain AHLs [10]. AHL-acylase AhlM is able to degrade AHLs and penicillin G, suggesting low substrate specificity [17]. The AiiC acylase from *Anabaena* can hydrolyze a set of AHLs that differ in the acyl chain length and substitution, although it shows preference for long-chain AHLs (more than C10-HSL) [54]. Such broad degradation ability is not usually observed in the other known AHL-acylases; instead, preference for one particular AHL or a specific group of AHLs is commonly observed. A genomic sequence analysis revealed two putative AHL acylases, HacA and HacB, in the *P. syringae* pathovar *syringae* B728a [21]. HacA is a secreted AHL-acylase degrading only long-chain AHLs (C > 8), and HacB is not the secreted AHL-acylase and degrades most AHLs. The targeted disruptions of *hacA*, *hacB* or both *hacA* and *hacB* do not alter endogenous 3-oxo-C6-HSL levels.

In identifying AHL-acylase Aac from *R. solanacearum* GMI1000, Chen *et al.* [38] showed that C7-HSL could be digested into HSL and heptanoic acid. The AHL-acylase exhibits activity against long-chain AHLs (C7-HSL, C8-HSL, 3-oxo-C8-HSL and C10-HSL) but not the short-chain AHLs (C4-HSL, C6-HSL and 3-oxo-C6-HSL). Park *et al.* [17] studied the substrate specificity of the AHL-acylase AhlM from *Streptomyces* sp. M664, which effectively degraded C8-HSL, C10-HSL and 3-oxo-C12-HSL with different acyl chain substitutions but exhibited relatively low activity on short-acyl-chain AHLs, such as C6-HSL and 3-oxo-C6-HSL, and did not degrade detectable amounts of C4-HSL. The AHL-acylase AhlM was effective in degrading AHLs with acyl chains longer than six carbons with or without substitution and was more active against unsubstituted AHLs than against 3-oxo-substituted AHLs.

The experimental data indicated that the *pvdQ* gene from *P. aeruginosa* PAO1 was sufficient but not necessary for long-acyl-chain AHL degradation because many *pvdQ* mutants retained the ability to degrade and utilize AHLs [20]. This finding suggested that *P. aeruginosa* encodes at least one additional AHL-acylase enzyme. Another *quiP* gene was identified from the *P. aeruginosa* genome [40]. The constitutive expression of QuiP in *P. aeruginosa* PAO1 resulted in the decreased accumulation of the 3-oxo-C12-HSL signal, indicating that QuiP is active against physiologically relevant concentrations of AHL produced by *P. aeruginosa*. QuiP is sufficient to catalyze the degradation of long- but not short-chain AHLs in *E. coli*. Differing from PvdQ, QuiP is able to degrade certain acyl-HSLs and cells with QuiP may utilize HSLs as growth substrate and quench QS in *P. aeruginosa*. The gene *pa0305* encodes penicillin acylase, whereas PA0305 can degrade AHLs with acyl side chains ranging from six to 14 carbons in length [67]. The overexpression of the *pa0305* gene in *P. aeruginosa* showed a significant reduction in the accumulation of 3-oxo-C12-HSL and the expression of virulence factors. The multiple AHL-degrading enzymes that occurred in a single strain add complexity to their functional roles. The phytopathogen *A. tumefaciens* harbors two AHL lactonases [68].

Most of the known AHL-lactonases hydrolyzing the lactone ring of AHLs do not display any preference for the length of the carbon acyl side chain attached to the lactone ring. The AHL-lactonase AidH from *Ochrobactrum* displays a strong hydrolyzing activity against C4-HSL, C6-HSL, C10-HSL, 3-oxo-C6-HSL and 3-oxo-C8-HSL [30]. Its lactonase activity greatly relies on Mn^2+^, although bioinformatic analyses did not reveal any potential metal ion-binding site on AidH. The AHL-lactonase AiiA from *Bacillus* displayed strong enzymatic activity toward AHLs varying in length and nature of the substitution at the C3 position of the acyl chain [69]. The amide group and ketone at the C1 position of the acyl chain of AHLs could be important structural features in enzyme-substrate interaction. The lactonase AiiA_B546_ from *Bacillus* sp. B546 has been confirmed with broad substrate specificity against C4-HSL, C6-HSL, C8-HSL, C10-HSL, C12-HSL, C14-HSL, 3-oxo-C6-HSL and 3-oxo-C8-HSL [70]. Cao *et al.* [71] found that the AiiA_AI96_ lactonase could degrade C4-HSL, C6-HSL, C7-HSL, C8-HSL, C10-HSL, C14-HSL, 3-oxo-C6-HSL, 3-oxo-C8-HSL, 3-oxo-C10-HSL, 3-oxo-C12-HSL and 3-oxo-C14-HSL. The AHL-lactonase QsdA from *Rh. erythropolis* confers the ability to inactivate AHLs with an acyl chain ranging from C6 to C14 with or without substitution at carbon 3 [33]. SsoPox as an AHL-lactonase from the archaeon *Sul. solfataricus* has preference for AHLs with acyl chain lengths of at least eight carbon atoms [72]. SsoPox can degrade C4-HSL and 3-oxo-C12-HSL, which are important for QS in the *P. aeruginosa* model system.

Functional metagenomics is a powerful technology to quickly identify a novel enzyme [73]. Very few studies have focused on the isolation of the novel AHL-degrading enzymes from metagenomes. The first study on the isolation of a metagenome-derived AHL-degrading enzyme was published in 2005 [74], and followed by another that led to the identification of the AHL-lactonase QlcA, an AiiA-like enzyme [59]. Subsequently, Bijtenhoorn *et al.* [64] reported the isolation and characterization of a novel hydrolase derived from a soil metagenome, designated as AHL-lactonase BpiB05, which is not similar to any known lactonase. It strongly reduces motility, pyocyanin synthesis and biofilm formation in *P. aeruginosa*. Another novel *est816* gene encoding an esterase was isolated from a Turban Basin metagenomic library and showed a broad substrate spectrum and high AHL-lactonase activity [75].

Most known AHL-lactonases are classified into the metallo-β-lactamases (MBL) superfamily, which has a conserved Zn^2+^ binding domain HXHXDH motif of metallo-hydrolytic enzymes. The active site of AHL-lactonase AiiA contains a dinuclear Zn^2+^ binding center bridged by an aspartate and oxygen species [76,77]. The AHL-lactonase AiiB from *A. tumefaciens* has an identical active site [78]. AHL-lactonase does not contain or require zinc or other metal ions for enzyme activity, although it carries an HXHXDH sequence [69,70]. This finding is true for the metagenome-derived enzymes that have been characterized recently [33,50,53]. Experimental data have suggested that AHL-lactonase is a highly specific enzyme and that ^106^HXDH^109^~H^169^ represents a novel catalytic motif that does not rely on zinc or other metal ions for activity [69]. The effects caused by metal ions differ between the AHL-lactonases and other MBLs, although AHL-lactonases have the typical MBL superfamily structure and contain the identical conserved residues and Zn ions.

The AHL-lactonase AiiA_B546_ from *Bacillus* sp. B546 was produced extracellularly in *Pichia pastoris* using 3-oxo-C8-HSL as the substrate [70]. Its molecular weight is 33.6 kDa with *N*-glycosylation. The AiiA_B546_ showed the optimal activity at pH 8.0 and 20 °C and had wide substrate specificity. When co-injected with *Aeromonas hydrophila* in common carp, AiiA_B546_ could decrease the mortality rate and delay the mortality of fish, suggesting a promising future application of AHL-lactonase in fish to control *Aer. hydrophila* disease by regulating its virulence. The AHL-lactonase AiiA_AI96_ from *Bacillus* sp. AI96 can maintain 100% of its activity at temperatures from 0 °C to 40 °C at pH 8.0 and shows broad-spectrum substrate specificity [71]. It is very stable at 70 °C and pH 8.0 for 1 h and resists digestion by proteases, which has not been found for all other *Bacillus* AHL-lactonase under the same conditions. When administered orally by fish feed supplementation, AiiA_AI96_ significantly attenuated *Aer. hydrophila* infection in zebra fish.

## 5. Molecular Phylogenesis of QQ Enzymes in the QS System

**Figure 4:**
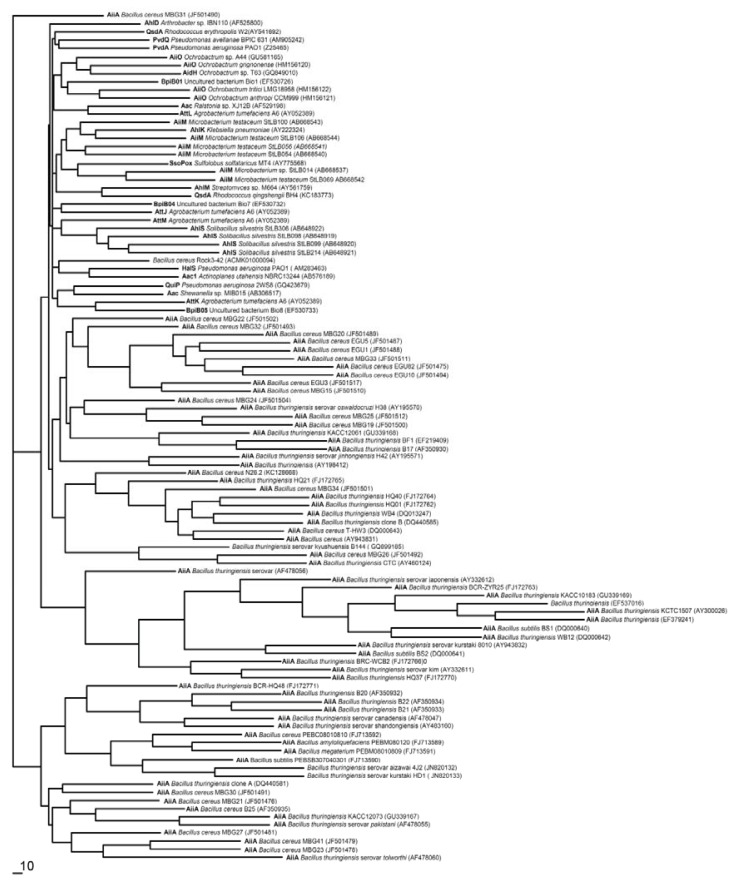
Phylogenetic tree showing the position of the known QQ enzymes among the different lactonase and acylase groups based on the comparison of gene sequences of QQ enzymes. The name of the organism from which it originates is supplied and the accession numbers of the QQ genes are indicated in parentheses. The scale bar represents 1% sequence dissimilarity.

Database searches for homologues of the characterized QQ enzymes have shown that relatives exist in a wide range of species, whereas the genes encoding AHL-lactonase have been found to be largely spread in microbes compared with genes encoding AHL-acylase. The phylogenetic position for AHL-lactonase and AHL-acylase was constructed using the neighbor-joining method shown in Figure 4 based on the QQ enzyme gene sequences.

According to the sequence similarity, lactonase can be divided into two groups. The first group contains a sole QsdA lactonase member, and all the other known lactonases, such as AiiA, are included in the second group. Phylogenetic analyses further divided these lactonases into two clusters, the AiiA-like cluster and the AttM-like cluster [44]. The AiiA-like cluster consists in all the AHL-lactonases from *Bacillus* and shares more than 90% identity at the amino acid level. The AttM-like cluster, including the AttM and AiiB lactonases from *A. tumefaciens* [15,28] and AhlD from *Arthrobacter* sp. IBN110 [18], shares 30%–58% homology in the peptide sequence and less than 25% identity with the AiiA-like cluster [44]. BpiB04 is a member of the glycosyl hydrolase family, and BpiB05 is a member of the dienelactone hydrolase family [53,64], although they are normally members of the MBL superfamily. The lactonase gene *aac* from *R. solanacearum* GMI1000 is considerably similar to that of the AHL-acylase from *Ralstonia* sp. XJ12B, with an 83% identity match and shared 39% identity with an aculeacin A acylase precursor from the gram-positive actinomycete *Actinoplanes utahensis* in the amino acid sequence [38].

## 6. Enzymatic Degradation of QS Signal Molecules in the Cell-Cell Signal Transduction Pathway

QS can be quenched by degrading AHL signal molecules using QQ enzymes to cause interference with the expression of AHL-regulated traits [19,79]. The QQ enzyme shows high specificity toward QS signal molecules but no influence on other molecules [69]. Some microbes not only produce QQ enzymes as a defense strategy against their competitors but also utilize AHL and its enzymatic degradation products as the sole carbon and nitrogen sources for cell growth [80]. When AHL-acylase from *Streptomyces* sp. was applied to a *P. aeruginosa* culture, a reduction of virulence factor production but not cellular growth was observed [17]. The *comamonas* strain D1 harboring AHL-acylase can enzymatically inactivate the QS signal molecule AHLs [23]. It degrades AHL with acyl-side chains ranging from four to 16 carbons with or without 3-oxo or 3-hydroxy substitutions. When co-cultured with other pathogens, some QS-dependent functions, such as violacein production by *C. violaceum* and pathogenicity and antibiotic production in *Pectobacterium*, can be quenched.

Recombinant *E. coli* with AiiA lactonase activity was shown to attenuate the pathogenicity of *E. carotovora* when co-cultured together [13]. The expression of *aiiA* in the insecticide *B. thuringiensis* could confer the strain with a strong biocontrol capacity against the AHL-dependent pathogen *E. carotovora* when co-inoculated with the pathogen [81].

The *aac* gene encodes an AHL-acylase from *R. solanacearum* [82]. Its heterologous expression in *C. violaceum* CV026 effectively inhibited violacein and chitinase activity, which were regulated by the QS mechanism, indicating that the acylase Aac could control AHL-dependent pathogenicity. The expression of the AHL-lactonase from *B. thuringiensis* in the phytopathogen *E. carotovora* resulted in substantially reduced levels of AHL via the enzymatic degradation of QS signal molecules, leading to decreased pectolytic enzyme activities, and attenuated *E. carotovora* disease symptoms on potatoes and cabbage [2].

To determine the capability of the QQ enzymes to block pathogenicity and toxoflavin production by the QS pathogen *Bur. glumae*, which causes rice grain rot, the AHL-lactonase gene *aiiA* was introduced into this bacteria [83]. The results showed that the AHL level in the transformants was reduced significantly and that the severity of the soft rot caused by *Pec. carotovorum* sp. *carotovorum* could be decreased when co-cultured with the recombinant *Bur. glumae*. The rice seedling or rice grain rot could not be shut down in the *aiiA*-transformant, suggesting that the gene *aiiA* encoding enzyme did not affect the virulence or toxoflavin production in *Bur. glumae*. Other types of QS signal molecules in addition to AHL-like molecules are presumed to occur in *Bur. glumae* that regulate virulence production.

The opportunistic pathogen *P. aeruginosa* can cause high mortality rates and typically occurs in immune-compromised patients and cases of hospital-acquired infections [84]. The strain *P. aeruginosa* PAO1 and its closely related pseudomonad are able to degrade and utilize AHL with long-chains (≥8 carbons) but not short-chains as the sole carbon and nitrogen sources for cell growth [20]. The QQ enzyme expressed in *P. aeruginosa* has been confirmed to reduce the accumulation of the long AHL signal 3-oxo-C12-HSL and prevent the accumulation of the short AHL signal C4-HSL, which results in a decrease in the swarming motility and virulence factor production [85]. The expression of the AHL-acylase *aiiD* in *P. aeruginosa* PAO1 changed the QS-regulated phenotypes, *i.e.*, attenuated its ability to produce elastase and pyocyanin, paralyze nematodes and form a biofilm [10]. When the AHL-acylase gene *pvdQ* was transformed in *P. aeruginosa* PAO1, the overproduced PvdQ was shown to be less virulent than the wild-type strain in a *Caenorhabditis elegans* infection model [86]. More than 75% of nematodes exposed to the transformed strain survived and continued to grow in a fast-acting paralysis assay when using this strain as a food source. Hypothetically, AHL-acylase enables *P. aeruginosa* PAO1 to modulate its own QS-dependent pathogenic potential.

A very limited number of QQ enzymes have been characterized, and future work should elucidate the diversity of this class of enzymes and its role in microbial cell-cell communication.

## 7. Application of Enzymatic Protection in Controlling Microbial Disease by Interfering with the QS System

With the emergence of antibiotic-resistant strains, the available options for treating bacterial infection are limited. The QQ strategy has been proposed as a sustainable therapy, considering its more limited selective pressure for microbial survival than antibiotic treatment. To date, two strategies have been developed as novel therapeutic tools for controlling plant diseases. (i) In a co-culture of the quorum quencher with QS-dependent pathogens in a heterologous system, the QQ enzymes produced by QQ microbes can limit or abolish the accumulation of QS signal molecules in the environment, resulting in a significant reduction in the QS-mediated phenotype. (ii) Plants engineered to produce QQ enzymes showed an elevated tolerance to the pathogen when challenged with the QS-mediated pathogen.

The first application of the QQ strategy in protecting against microbial infection was conducted by Dong *et al.* [2], in which the *aiiA* gene was transformed into the phytopathogen *E. carotovora* to attenuate its decay phenotype in Chinese cabbage. The AHL-lactonase was successfully expressed in other pathogens, such as *E. amylovora*, *P. aeruginosa* PAO1, *Bur. cepacia* and *Pic. Pastoris*, to reduce their virulence [70,79,85,87]. The transformation of *aidH* into *P. fluorescens* resulted in a decrease in biofilm formation and transformation into *Pec. carotovorum* resulted in the attenuation of soft-rot disease symptoms [30]. Plants engineered to express AHL-lactonase demonstrated a capability for substantially enhanced resistance to *E. carotovora* infection [88]. When infected with *E. carotovora*, transgenic tobacco or potato developed no symptoms or small maceration areas, depending on the cell density of the inoculums.

Recently, we isolated a QQ bacterium *B. marcorestinctum* from soil that strongly quenches the AHL QS signal [11]. When *B. marcorestinctum* was applied to sliced potato tubers with *Pec. carotovorum*, the soft rot symptoms caused by *Pec. carotovorum* were effectively attenuated, suggesting that the co-culture of QQ microbes with a QS-mediated pathogen could be used for preventing a host for QS microbial infection by inactivating AHL autoinducing activity.

A biofilm is a matrix-enclosed microbial aggregation that adheres to a biological or non-biological surface [89]. The QS signal molecule has been shown to mediate biofilm formation to protect against antibiotics [90]. Biofilm infection is difficult to eradicate because of its much better protection against macrophages and antibiotics compared with free living cells. A well-known and extensively studied biofilm is the attachment of bacteria to teeth to form dental plaque. Paul *et al.* [91] showed the potential of acylase I to quench the biofilm formation by environmental strains of bacteria. Acylase I was found to reduce biofilm formation by *Aer. hydrophila* and *P. putida* on borosilicate (36% and 23%, respectively), polystyrene (60% and 73%, respectively) and a reverse osmosis membrane (20% and 24%, respectively).

Membrane bioreactors have gained increasing attention in engineering applications for wastewater treatment; however, the biofoul on membranes caused by biofilm growth decreases membrane permeability and lifespan [92]. Experimental data showed that the QQ enzyme could influence sludge characteristics and biofouling without affecting pollutant degradation, suggesting that the QQ strategy is a promising approach to mitigate biofouling in membrane filtration processes [93]. The immobilized acylase weakened the ability to form a biofilm and enhanced membrane filterability. The reduction in biofouling by QQ enzymes was found to be reversible, and the subsequent membrane performance was not affected when the QQ ceased. Yeon *et al.* [94] used AHL-acylase attached to a magnetic carrier to inhibit QS in MBR for advanced wastewater treatment. The authors found that it reduced biofouling effectively and enhanced membrane permeability. Kim *et al*. [95] coupled the AHL-acylase directly on a nanofiltration membrane surface for wastewater treatment. These membranes could interfere with the QS system in the membrane biocake and reduce biofouling. The application of QQ is a promising alternative for mitigating membrane biofouling [96].

The QQ strategy plays an important role in preventing microbial disease and affecting beneficial microbes. Most plant rhizobacteria have been found to use AHLs as signal molecules to mediate functional activities such as triggering systemic resistance in the host and producing antifungal compounds that are essential to their survival or the establishment of beneficial interactions with the plant [97]. AHL-lactonase AiiA is necessary for rhizosphere colonization and its survival in the soil [98]. A mutant strain defective in AHL-lactonase AiiA was unable to successfully colonize the tested rhizosphere, and its viability was significantly reduced. This finding suggested that the QQ enzyme was involved in microbial competitiveness in the rhizosphere and helped bacteria survive on the plant root. The introduction of the *aiiA* gene from *Bacillus* led to a significant decrease in the number of nodules induced on *Medicago truncatula* and to strong modification of AHL-mimicking compounds [99]. This finding strongly indicates that the effect of the QQ procedures on non-targeted bacterial populations should not be neglected. Specific efforts should be targeted toward the identification of highly specific QQ enzymes to reduce the risks in applying QQ to beneficial microbes.

## 8. Future Works

In a brief time, numerous QQ phenomena have been observed, and QQ strategies have been tested with promising results. The degradation of the QS signal itself is not sufficient to completely diminish the QS activities in a number of cases. The lactonase treatment resulted in only a 73.42% reduction in biofilm formation, although its antibiotic susceptibility was increased to 3.3-fold the minimum biofilm eradication concentration for ciprofloxacin [100]. Strain *B. cereus* U92 reduced the frequency of Ti-plasmid conjugal transfer in *A. tumefaciens* by approximately 99% in binary cultures, alleviated QS-regulated crown gall incidence on tomato roots (less than 90%) and attenuated *Pectobacterium* soft rot on potato tubers (less than 60%) in quantitative experiments [101]. More than one AHL-based QS system occurs in the identical isolate, which are all involved in the global regulation of biocontrol-related traits in the QS network [102,103]. Some QQ enzymes showed high substrate specificity. These enzymes present a challenge in developing approaches that target a broad range of signaling molecules. Further investigation should focus on studying the substrate specificity of QQ enzymes in the hierarchical QS system and searching for the universal QQ enzymes that target a broad range of AHLs for efficient blockade of QS activity. From a biocontrol point of view, a combination of the QQ approach with other treatments, such as antibiotics, to obtain a synergistic effect is a potential strategy that could potentially increase the susceptibility of bacteria to antibiotic treatment.

## 9. Conclusions

QQ enzymes have been identified in QS- and non-QS microbes, including AHL-lactonase, AHL-acylase, oxidoreductase and paraoxonase. AHL-lactonase is widely conserved in a range of bacterial species and has variable substrate spectra. The QQ enzymes that occurred in non-QS microbes could degrade neighboring bacteria to protect against microbial infection and metabolize the QS signal molecules for cell growth. The existence of QQ enzymes in QS microbes attenuates QS efficiency and blocks unnecessary gene expression and pathogenic phenotypes to act as a defensive mechanism. The QQ enzymes could reduce the virulence of QS microbes without resulting in antibiotic-resistance. The external addition of the QQ enzymes may represent a novel general antibacterial therapy and highlights the potential value of QQ enzymes to protect against bacterial infection.

## Figures and Tables

**Table 1:** Quorum quenching (QQ) enzymes involved in the degradation of the quorum sensing (QS) signals AHLs.

Enzyme	Host	Substrate	References
**AHL lactonase**			

	*Bacillus* sp. 240B1	C6-10-HSL	[2]
	*Bacillus cereus* A24	AHL	[12]
AiiA	*Bacillus mycoides*	AHL	[12]
	*Bacillus thuringiensis*	AHL	[13]
	*Bacillus anthracis*	C6, C8, C10-HSL	[14]
AttM	*Agrobacterium tumefaciens*	3-oxo-C8-HSL, C6-HSL	[28]
AiiB	*Agrobacterium tumefaciens* C58	Broad	[15]
AiiS	*Agrobacterium radiobacter* K84	Broad	[58]
AhlD	*Arthrobacter* sp. IBN110	Broad	[18]
AhlK	*Klebsiella pneumoniae*	C6-8-HSL	[18]
QlcA	*Acidobacteria*	C6-8-HSL	[59]
AiiM	*Microbacterium testaceum* StLB037	C6-10-HSL	[31]
QsdA	*Rhodococcus erythropolis* W2	C6-14-HSL with or without C3-substitution	[33]
AidH	*Ochrobactrum* sp. T63	C4-10-HSL	[30]
DlhR, QsdR1	*Rhizobium* sp. NGR234	nd.	[49]
AhlS	*Solibacillus silvestris* StLB046	C6-HSL, C10-HSL	[32]
SsoPox	*Sulfolobus solfataricus* strain P2	C8-12-HSL	[52,60]
	*Rhodococcus* sp.	Broad	[16]
GKL	*Geobacillus kaustophilus* strain HTA426	C6-12-HSL	[61]
PPH	*Mycobacterium tuberculosis*	C4, C8, C10-HSL,	[62]
MCP	*Mycobacterium avium* subsp. *paratuberculosis*	C7-12-HSL	[63]
BpiB01, BpiB04, BpiB05, BpiB07	Soil metagenome	3-oxo-C8-HSL	[53,64]
QlcA	Soil metagenome	C6-10-HSL	[59]

**AHL acylase**			

AiiD	*Ralstonia eutropha*	C8-12-HSL	[10]
PvdQ	*Pseudomonas aeruginosa*	C7-12-HSL with or without C3-substitution	[19,20]
QuiP	*Pseudomonas aeruginosa*	C7-14-HSL with or without C3-substitution	[40]
AiiC	*Anabaena* sp. PCC 7120	Chain length more than C10	[54]
AhlM	*Streptomyces* sp. M664	Chain length more than C8	[17]
Aac	*Ralstonia solanacearum*	Chain length more than C6	[38]
	*Shewanella* sp. MIB015	Broad but prefer long chain	[65]
HacA	*Pseudomonas syringae*	C8,C10, C12-HSL	[21]
HacB	*Pseudomonas syringae*	C6-12-HSL with or without C3-substitution	[21]
	*Variovorax* sp.	Broad	[42]
	*Variovorax paradoxus*	Broad	[9]
	*Tenacibaculum maritimum*	C10-HSL	[66]
	*Comomonas* sp. D1	C4-16-AHL with or without C3-substitution	[23]
	*Rhodococcus erythropolis* W2	C10-HSL	[22]

**Oxidoreductase**			

P450BM-3	*Bacillus megaterium* CYP102 A1	C12-20-HSL(ω-1, ω-2, ω-3 hydroxylated)	[55]

nd, not determined.

## Abbreviations

*B.*: *Bacillus*
*E.*: *Erwinia*
*Pec.*: *Pectobacterium*
*V.*: *Variovorax*
*R.*: *Ralstonia*
*A.*: *Agrobacterium*
*Rh.*: *Rhodococcus*
*P.*: *Pseudomonas*
*K.*: *Klebsiella*
*M.*: *Microbacterium*
*S.*: *Solibacillus*
*C.*: *Chromobacterium*
*Bur.*: *Burkholderia*
*Sul.*: *Sulfolobus*
*Aer.*: *Aeromonas*
*Pic.*: *Pichia*
*Sta.*: *Staphylococcus*
C4-HSL: *N*-butanoyl-l-homoserine lactone
C6-HSL: *N*-hexanoyl-l-homoserine lactone
C7-HSL: *N*-heptanoyl-l-homoserine lactone
C8-HSL: *N*-octanoyl-l-homoserine lactone
C10-HSL: *N*-decanoyl-l-homoserine lactone
C12-HSL: *N*-dodecanoyl-l-homoserine lactone
C14-HSL: *N*-tetradecanoyl-l-homoserine lactone
3-oxo-C6-HSL: *N*-(3-oxohexanoyl)-l-homoserine lactone
3-oxo-C8-HSL: *N*-(3-oxooctanoyl)-l-homoserine lactone
3-oxo-C10-HSL: *N*-(3-oxodecanoyl)-l-homoserine lactone
3-oxo-C12-HSL: *N*-(3-oxododecanoyl)-l-homoserine lactone
3-oxo-C14-HSL: *N*-(3-oxotetradecanoyl)-l-homoserine lactone
